# Hybrid Illumina-Nanopore assembly improves identification of multilocus sequence types and antimicrobial resistance genes of Staphylococcus aureus isolated from Vermont dairy farms: comparison to Illumina-only and R9.4.1 nanopore-only assemblies

**DOI:** 10.1099/acmi.0.000766.v3

**Published:** 2024-03-25

**Authors:** Ashma Chakrawarti, Korin Eckstrom, Pheobe Laaguiby, John W. Barlow

**Affiliations:** 1 Department of Animal and Veterinary Sciences, University of Vermont, Burlington, VT, USA; 2 Department of Microbiology and Molecular Genetics, Robert Larner, M.D. College of Medicine, University of Vermont, Burlington, VT, USA; 3 Advanced Genome Technologies Core, Vermont Integrative Genomics Resource, The Robert Larner, M.D. College of Medicine, University of Vermont, Burlington, VT, USA

**Keywords:** folp gene, hybrid assembly, MLST, phenotype, resistance genes, spa type, whole-genome sequencing (WGS)

## Abstract

Antimicrobial resistance (AMR) in *Staphylococcus aureus* is a pressing public health challenge with significant implications for the dairy industry, encompassing bovine mastitis concerns and potential zoonotic threats. To delve deeper into the resistance mechanisms of *S. aureus*, this study employed a hybrid whole genome assembly approach that synergized the precision of Illumina with the continuity of Oxford Nanopore. A total of 62 isolates, collected from multiple sources from Vermont dairy farms, were sequenced using the GridION Oxford Nanopore R9.4.1 platform and the Illumina platform, and subsequently processed through our long-read first bioinformatics pipeline.

Our analyses showcased the hybrid-assembled genome’s superior completeness compared to Oxford Nanopore (R9.4.1)-only or Illumina-only assembled genomes. Furthermore, the hybrid assembly accurately determined multilocus sequence typing (MLST) strain types across all isolates. The comprehensive probe for antibiotic resistance genes (ARGs) using databases like CARD, Resfinder, and MEGARES 2.0 characterized AMR in *S. aureus* isolates from Vermont dairy farms, and revealed the presence of notable resistance genes, including beta-lactam genes *blaZ*, *blaI*, and *blaR*. In conclusion, the hybrid assembly approach emerged as a tool for uncovering the genomic nuances of *S. aureus* isolates collected from multiple sources on dairy farms. Our findings offer a pathway for detecting AMR gene prevalence and shaping AMR management strategies crucial for safeguarding human and animal health.

**Impact Statement**

Antimicrobial resistance (AMR) is a pressing global concern, threatening human health and livestock industries. Globally, dairy farmers grapple with AMR strains of bacteria like *Staphylococcus aureus*, which both harm livestock and have potential human health implications. Our study delves into this issue by leveraging advanced genetic sequencing methods, providing an unprecedented look into the genome of *S. aureus* strains from Vermont dairy farms. Our hybrid assembly approach paints a clearer picture than earlier techniques, refining our understanding of resistance mechanisms and genetic diversity. While rooted in Vermont’s dairy industry context, this paper holds wide-reaching implications. An improved understanding of these bacteria can be used to strengthen strategies to mitigate their effects on dairy farms and address potential zoonotic threats. This research is an incremental advancement in tackling global AMR issues in dairy industries. It’s an invitation for stakeholders, from veterinarians to public health officials, to leverage this refined knowledge, ultimately benefiting livestock welfare and human health.

## Data Summary

All sequence data was submitted to the National Centre for Biotechnology Information (NCBI) sequence read archive (SRA) under the project number PRJNA983748, biosample accession number SAMN36020147 to SAMN36020208 (Table S1).

## Introduction

*S. aureus* is a significant mastitis pathogen of dairy cattle, negatively affecting animal welfare and milk production [1]. It is also a zoonotic pathogen capable of crossing species barriers and causing infections in humans and animals [2]. This issue is compounded by the presence of antimicrobial resistant *S. aureus* strains on dairy farms, making it imperative to accurately understand the genomic epidemiology of this bacterium [1]. Hence, investigating resistant *S. aureus* strains in dairy settings is crucial for reducing the potential animal and human health risks.

Over recent years, several methods such as multilocus sequence typing (MLST) and staphylococcal protein A (spa) typing have been utilized to study molecular epidemiology and typing of *S. aureus* human and animal strains [3,5]. Despite their utility, these techniques are labour intensive and often fail to reveal intricate genetic differences between strains [6,7]. Whole-genome sequencing (WGS) offers in-depth comprehensive genotype information, including whole genome MLST and spa typing by WGS, enhancing our understanding of strain diversity and the genetic determinants of antimicrobial resistance [7,9].

Antimicrobial resistance (AMR) poses a global health challenge, significantly impacting both the human and livestock health [10,11]. *Staphylococcus aureus*, known for its proclivity to acquire resistance, is a prime example of an AMR pathogen that is adaptable to multiple host species [12,13]. It presents a substantial concern in the dairy industry, as it not only leads to economic losses from bovine mastitis, but also poses a potential zoonotic threat [14,15]. These multifaceted aspects of AMR necessitate the use of advanced molecular tools to gain a comprehensive understanding of resistance genes and mechanisms in *S. aureus* [8]. AMR gene detection and strain identification from genome analysis demands accurate and cost-effective high-throughput sequencing and downstream bioinformatics. The choice of sequencing and assembly methods significantly influences the detection and localization of resistance genes, underscoring the importance of strategically selecting sequencing platforms and assembly approaches [16,19].

Next-generation sequencing technologies like Illumina provide high-accuracy, high-throughput data. However, Illumina’s short read length often results in fragmented genome assemblies, creating challenges for downstream analyses [19,20]. Third-generation sequencing technologies like Oxford Nanopore address this limitation by providing longer reads, but these often come with a higher error rate [21,22]. To mitigate the limitations of both short and long-read sequencing, our study applies a hybrid assembly approach, combining the accuracy of Illumina and the continuity of Oxford Nanopore. The process begins with long-read assembly using Oxford Nanopore data to capture the genome’s overall structure. Subsequently, Illumina data is used for polishing, correcting errors, and enhancing assembly quality. This integrated approach seeks to harness the strengths of each sequencing technology, leading to a more reliable and comprehensive genome assembly [17,19].

By leveraging cutting-edge genomic tools and methods, this study aims to identify strain types and AMR genes of *S. aureus* isolated from Vermont dairy farms using a hybrid assembly approach. The overarching goal is to advance understanding of *S. aureus* genomic diversity and predict AMR profiles, ultimately informing strategies for *S. aureus* control and AMR management in both animal and human health.

## Methods

### Provenance of isolates

Isolates were collected during previous observational field studies of *S. aureus* epidemiology on Vermont dairy farms and stored frozen in the isolate collection of the University of Vermont Quality Milk Research Laboratory. Sixty-two isolates collected from different sources from Vermont dairy farms were included in this study and were selected to represent a variety of strains. Briefly, the isolates were recovered from frozen stocks and included 24 human hand and nasal swabs isolates, 24 bulk tank milk sample isolates, five cattle hock (tarsus) skin lesion swab isolates and ten cow quarter milk sample isolates collected from 23 Vermont dairy farms between 2003 and 2020. The details of isolates are provided in supplementary files (Table S1).

### DNA extraction

Isolates were recovered from frozen stocks by inoculation on Tryptic Soy Agar with 5 % sheep blood, to obtain single pure colonies which were then transferred to Tryptic Soy Broth and incubated overnight at 37 °C. High molecular weight genomic DNA was extracted using Nanobind CBB Big DNA kit from Circulomics following the extraction protocol for Gram-positive bacteria-HMW. The concentration and quality of extracted DNA were assessed using Nanodrop and Qubit 4.0 fluorometer. The extracted DNA was then stored at 4 °C in TE buffer until library preparation and sequencing.

### Sanger sequencing of MLST amplicons

All isolates were strain typed using traditional MLST methods as described by Enright *et al*. for amplification and Sanger (di-deoxy chain termination) sequencing of internal fragments of seven house-keeping genes [3]. MLST relies on single nucleotide polymorphisms (SNPs) to define alleles for each of the seven genes, and the allelic profile defines the sequence type (ST) of an isolate. Amplicons were sequenced in both directions and consensus alignments of both forward and reverse chromatogram trace files used to define alleles. Alleles, STs, and clonal complexes (CC) were determined using the MLST database (https://pubmlst.org/organisms/staphylococcus-aureus). DNA sequence trace chromatograms were submitted to the database curator for assignment of any novel alleles and STs (allelic profiles).

### Library preparation and whole genome sequencing

The extracted DNA samples were submitted to the University of Vermont Advanced Genome Technologies Core facility for library preparation and long read sequencing using GridION Oxford Nanopore platform (ONT) R9.4.1. Library preparation was performed using different kits SQK-LSK109, EXP-NBD104, EXP-NBD114 and EXP-NBD196 since the sequencing of these samples was done in multiple batches. At least 20 multiplexed library samples were sequenced in a single flow cell (FLO_MIN106) for 72 h. High accuracy basecalling was performed using Guppy v4.0.11 and reads were exported as FASTQ files. For Illumina paired end sequencing, the extracted DNA samples were first diluted to 25 ng µl^−1^ each and used for library preparation using SeqOnce RhinoSeq Library prep kit and Nextera XT 96 sample kit following manufacturer’s instructions. The concentration and quality of the prepared libraries were determined using Bioanalyzer. Finally, each library DNA of 2–4 ng µl^−1^ was submitted for 150 bp paired end short read sequencing using MiniSeq Illumina platform. After basecalling, paired end reads were exported as zipped FASTQ files.

### Genome assembly and annotation

**Fig. 1.**
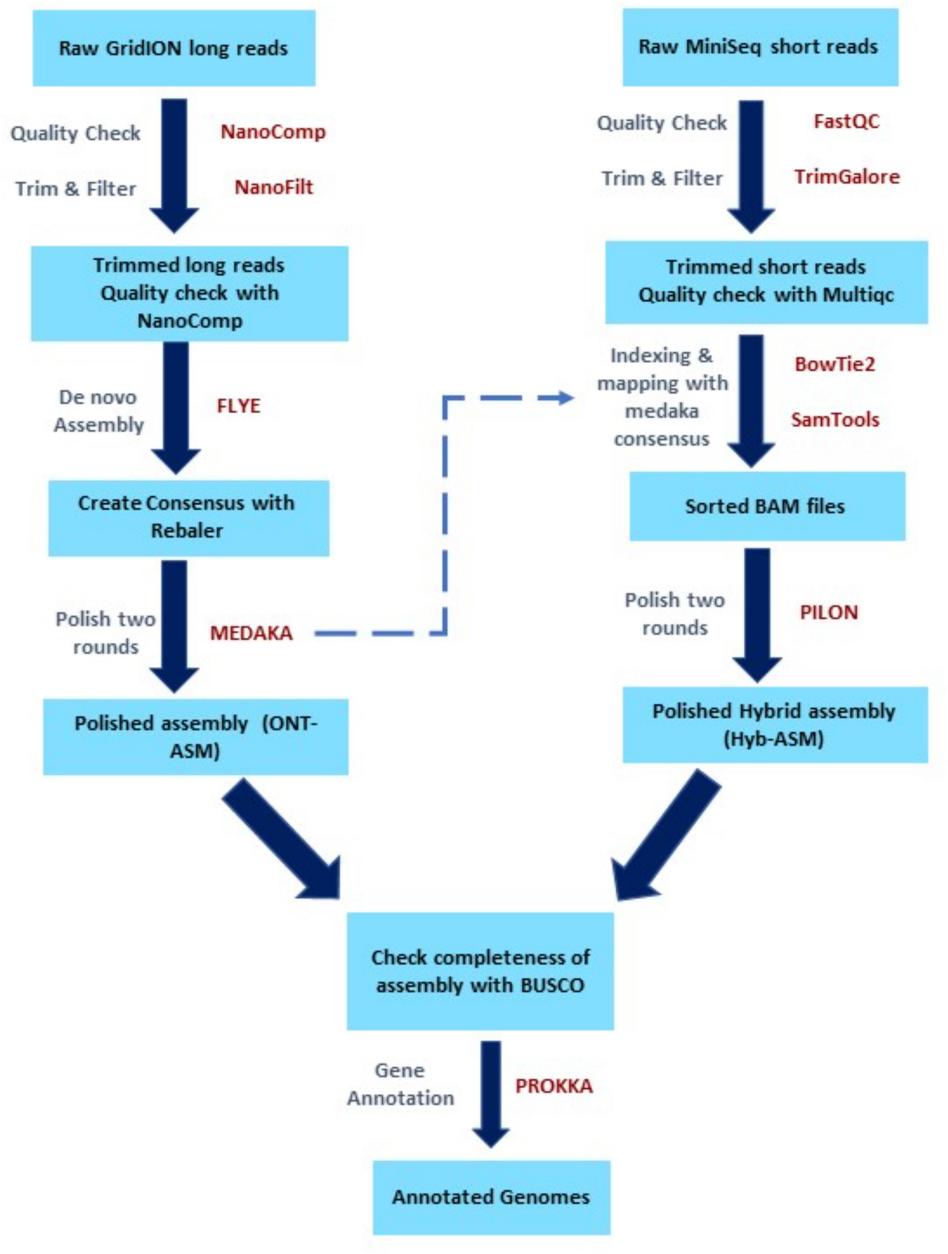
In-house bioinformatics pipeline for hybrid assembly and annotation of *S. aureus* genomes.

An in-house bioinformatics pipeline was constructed for *de novo* assembly of genomes (Fig. 1). In brief, the long-read assembled genomes were polished using their respective Illumina short reads to generate single contigs for each genome.

Nanocomp v1.13.0 was used to determine the quality of ONT long reads [23]. NanoFilt v2.7.1 with parameters l ‘1000’ and q ‘10’ was used to trim unwanted reads. *De novo* assembly of trimmed ONT reads was performed with Flye v2.3.1 assembler using genome size 2.8Mbps [24]. The assembled genomes were then polished using Rebaler v0.2.0 [25] and Medaka v1.0.3 [26] and identified as ONT-only assembled genomes (ONT-Asm).

For Illumina short reads, raw reads were checked for their quality using FastQC v0.11.9 [27]. The paired end raw reads were trimmed with TrimGalore v0.6.7 [28] with parameter q20. After trimming, the quality of trimmed reads was again checked using Multiqc v1.9 [29]. For hybrid assembly, the medaka polished genome assembly fasta files were indexed using Bowtie2 v2.4.2 [30] and mapped with short reads using Samtools v1.9 [31]. The generated bam files were then again polished using Pilon v1.23 [32] to obtain polished hybrid-assembled genomes (Hyb-Asm). Unicycler v0.5.0 [33], with default parameters, was used to assemble the Illumina short reads to generate Illumina-only assembled genomes (Illu-Asm). All three types of genome assemblies for 62 genomes were checked for their completeness and quality using BUSCO v4.1.2 [34]. The genome annotation was performed with PROKKA v1.14.6 [35] using *Staphylococcus* database.

### *In silico* multilocus sequence typing, spa types and detection of resistance genes

Whole genome multilocus sequence types (wgSTs) were predicted *in silico* from the annotated genomes using mlst v2.19.0 [36,37] and spa types (t) were predicted using spaTyper v1.0 webserver from Centre of Genomic Epidemiology (https://cge.cbs.dtu.dk/services/spatyper/) [38] for the three different genome assemblies of all 62 genomes. ARGs were detected using ABRicate v1.01 [39] utilizing the Comprehensive Antibiotic Resistance Database (CARD) [40], Resfinder [41,42], NCBI AMRFINDERplus [8], MEGARES 2.0 [43] and ARG-ANNOT [44].

### Antimicrobial susceptibility phenotypic analysis

Antimicrobial sensitivity was assessed using both agar disc diffusion (DD) and broth microdilution techniques, adhering to the standards set by the Clinical Laboratory Standards Institute (CLSI) [45]. For the broth microdilution tests, a commercial 96-well plate (Sensititre Mastitis MIC plates, CMV1AMAF, Trek Diagnostic Systems) was used to test susceptibility to ampicillin, cephalothin, ceftiofur, erythromycin, oxacillin (with 2 % NaCl), penicillin, penicillin/novobiocin, pirlimycin, sulfadimethoxine, and tetracycline. The disc diffusion method utilized commercially sourced discs to evaluate susceptibility to ampicillin, erythromycin, ceftiofur, tetracycline, oxacillin, amoxicillin/clavulanic acid, cefoxitin, clindamycin, enrofloxacin, gentamicin, lincomycin, vancomycin, cefazolin, tilmicosin, and sulfamethoxazole/trimethoprim. Quality control for these assays was maintained using *S. aureus* ATCC 25923 for disc diffusion and ATCC 29213 for broth microdilution. The outcomes from both methods were analysed in line with the CLSI recommendations [45]. Because the *S. aureus* isolates used in this study were collected from both humans and cattle, we believe it is noteworthy that zone diameter and MIC breakpoints in the CLSI veterinary standard for antimicrobial agents are derived from human data with some exceptions where data are available for mastitis isolates from cattle (e.g. ceftiofur, penicillin/novobiocin, and pirlimycin) [45].

### Data availability

The raw reads from ONT and Illumina for all 62 genomes are available under NCBI Bioproject accession number PRJNA983748 (Table S1).

## Results

### Basic statistics of Oxford Nanopore and Illumina reads

The average sequencing depth for the ONT reads before trimming was 178×, with an average read quality of 13.3. After trimming, the average sequencing depth for all 62 genomes was 146.52×, with an average read quality of 14. The average sequencing depth for the Illumina reads was 27×, with an average read quality of 36. The detail of sequencing reads quality and other parameters are provided in supplementary files (Table S1).

### Genome assembly and annotation

**Fig. 2.**
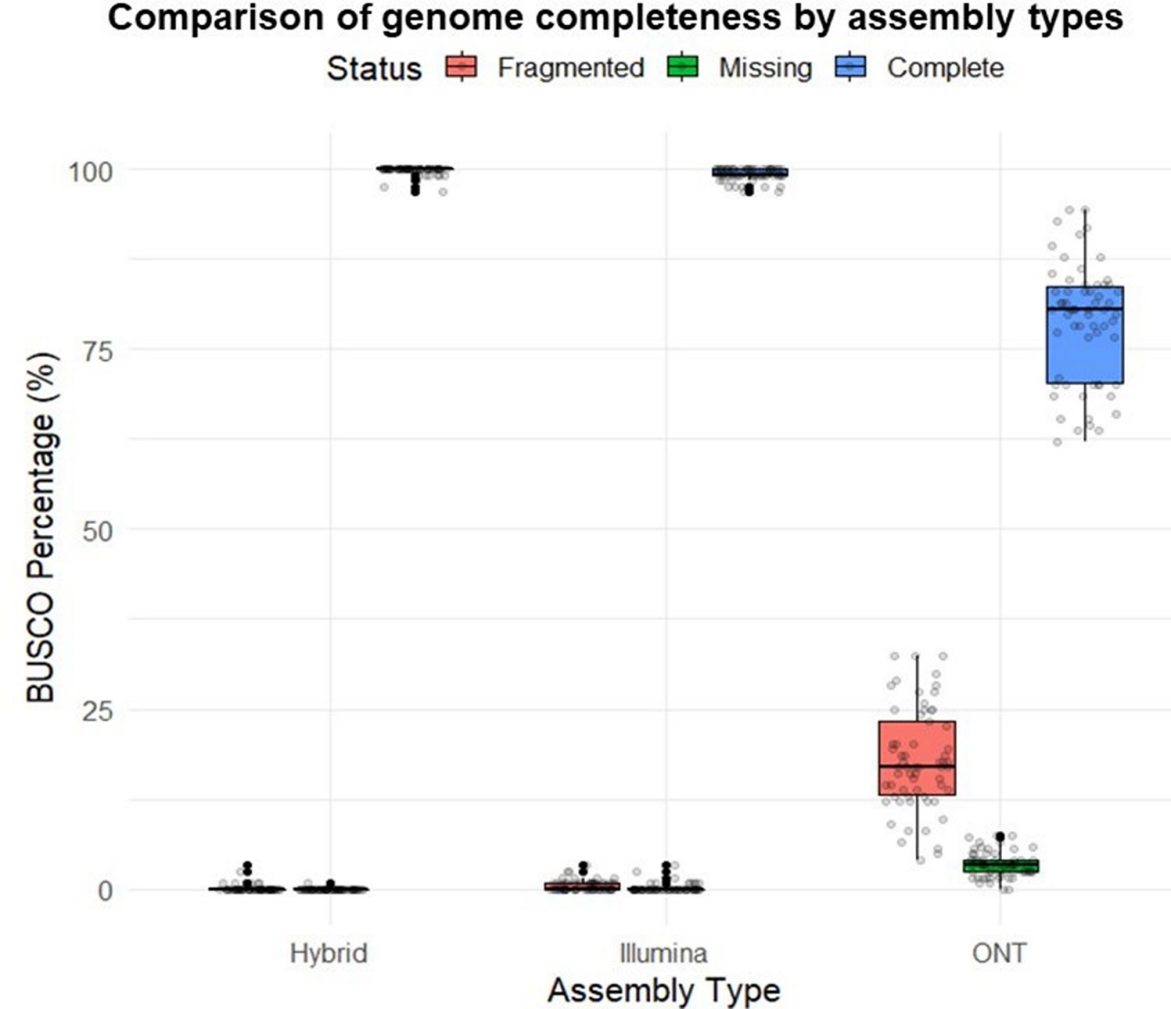
Comparison of genome completeness using BUSCO for different assembly methods. A boxplot showing percentage of fragmented, missing, and complete genes using three different genome assembly obtained from BUSCO.

The genome assembly completeness was assessed using BUSCO, and the results showed that the hybrid-assembled genome (Hyb-Asm) had better completeness than the ONT-only assembled genome (ONT-Asm) and the Illumina-only assembled genome (Illu-Asm). Specifically, the BUSCO analysis indicated that the Hyb-Asm and Illu-Asm genomes had an average completeness of 99.8 and 99.3 %, respectively, with a missing rate of 0.02 and 0.2 %, respectively. In contrast, the ONT-Asm genome had a lower completeness percentage, with 17.8 % of genes being fragmented and only 78.75 % being complete, and a missing rate of 3.36 % for BUSCO genes (Fig. 2).

The number of contigs greater than 500 bp in the final assembly of ONT-Asm and Hyb-Asm genomes were the same. Comparing the number of contigs greater than 500 bp between Hyb-Asm and IIu-Asm genomes, revealed a higher number of contigs in the Illumina approach. The average number of contigs for the Illumina method was 206, substantially exceeding the hybrid method’s average of 1.7. Similarly, the median number of contigs for Illumina was 192.50, in contrast to just 1.00 for the hybrid approach. The hybrid assembly method resulted in the most complete and accurate genome assembly. Detailed statistics for BUSCO analysis and number of contigs are provided in Table S2 and Fig. S2, respectively.

### Strain typing

#### Multilocus sequence typing (MLST)

The wgST of each of the 62 assembled genomes was determined using the three different assembly methods, and results were compared to the alleles and STs determined by the traditional Sanger sequencing method. We defined the alleles and STs determined by Sanger sequencing as the gold standard for comparison to wgST predictions from the assembled genomes. The hybrid assembly method (Hyb-Asm) was successful in accurately determining the MLST type for all isolates, (100 % concordance of wgST with the traditional MLST typing method). In contrast, the Illumina-only assembly method (Illu-Asm) provided the correct MLST for 59 (95 %) isolates, while the ONT-only assembly method (ONT-Asm) provided the correct MLST for only 42 (68 %) isolates (Table S3). From the ONT-Asm assemblies, one or more alleles were predicted as ‘undetermined’ for 19 of the isolates and one isolate was incorrectly typed.

Upon further investigation, it was found that the nucleotide sequence of five housekeeping genes (*pta, yqil, tpi, gmk, arcC*) in the ONT-Asm genomes of the undetermined isolates contained unwanted 'A' bases in polyA regions, gaps, and mismatches, which led to the undetermined MLST types predicted from the ONT-Asm assemblies. For the incorrectly typed isolate, the discrepancy was due to predicting an incorrect allelic number of the *arcC* gene. The Illu-Asm method was unable to determine the MLST type for three out of the 62 isolates, possibly due to suboptimal input data quality, leading to the missing hits for the *pta* and *glpF* alleles in the results. Additional details on the errors in MLST of these isolates can be found in Table S4. Overall, the results suggest that the hybrid assembly method produced the most accurate and reliable results for determining the MLST types of these *S. aureus* isolates.

#### Spa typing

The spa typing results were concordant between Hyb-Asm and ONT-Asm genomes. However, Illu-Asm genomes were unable to provide concordant spa types for 19 isolates. The detailed results of spa typing using the three different assembly methods are provided in supplementary materials (Table S5), and the specific spa type errors of predicted repeats are shown in Table S6.

### AMR genes identification and phenotypic antimicrobial susceptibility testing results

**Fig. 3.**
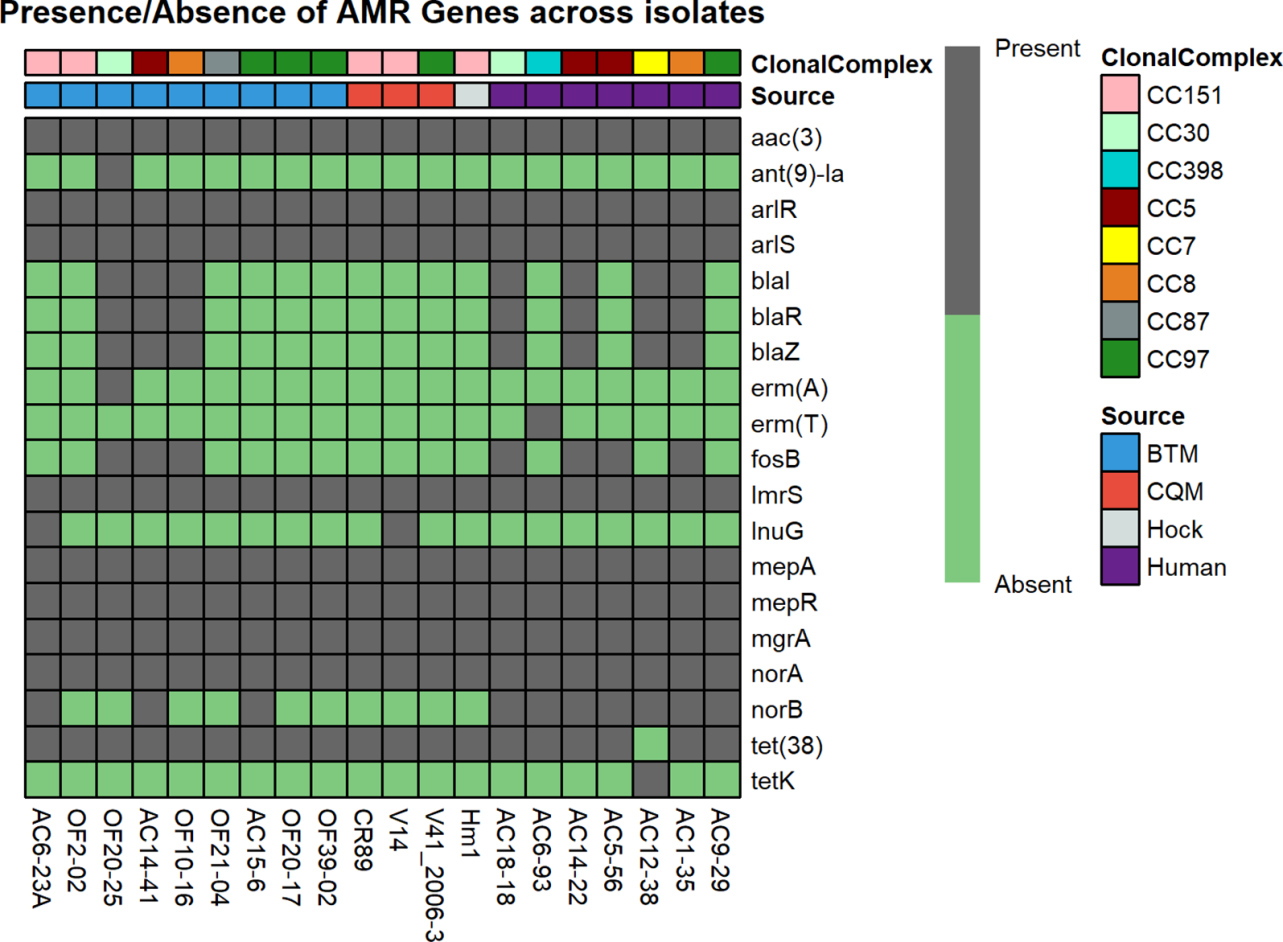
Presence/absence heatmap of AMR genes in *S. aureus* genomes representative of their source of isolation and clonal complex.

The genome assembly from all three methods gave near identical results for presence of AMR genes. The AMR genes identified from WGS of 62 isolates were concordant with available phenotypic antibiotic sensitivity tests results. In total, WGS predicted the presence of 19 AMR genes from seven drug classes, including some genes that confer resistance against fosfomycin, fluoroquinolones, phenicols, and streptogramins for which phenotypic tests were not performed. WGS also predicted point mutations for certain antibiotics like sulfadimethoxine and fosfomycin. Twenty isolates representing the three sources of isolates and eight clonal complexes show the 19 different AMR genes in a presence/absence heatmap (Fig. 3). A corresponding figure that includes all 62 isolates can be found in the supplementary materials (Fig. S1).

For the 17 drug classes where we conducted susceptibility testing there was agreement between observed phenotypic resistance and gene presence, except for tetracycline susceptibility (Table 1). The comprehensive dataset for the phenotypic antimicrobial susceptibility testing (AST) is available in the supplementary section (Table S7).

**Table 1.** Number of isolates stratified by presence and absence of resistance genes in relation to susceptibility phenotype by drug class

	Phenotype: Resistant (R)		Phenotype: Susceptible (S)	
**Drug classes/ genes**	Gene present	Gene absent	Gene present	Gene absent
**Beta lactams (*blaZ***)	28	0	0	34
**Cephalothin**	0	0	0	62
**Cefoxitin**	0	0	0	62
**AmoxyClav**	0	0	0	62
**Erythromycin**	8	0	0	54
**Gentamicin**	0	0	0	62
**Tetracycline**	3^a^	0	59^b^	0
**Ceftiofur**	0	0	0	62
**Pirlimycin**	5	0	0	57
**Oxacillin**	0	0	0	62
**PenNov**	0	0	0	62
**Clindamycin**	0	0	0	62
**Vancomycin**	0	0	0	62
**Enrofloxacin**	0	0	0	62
**Lincomycin**	5	0	0	57
**Cefazolin**	0	0	0	62
**Tilmicosin**	0	0	0	62

aa*tet(K)*, b*tet(38*).

#### Beta lactam resistance

The beta lactam resistance genes identified from these isolates are *blaz*, *blaI* and *blaR*. Twenty-six genomes had all three genes and were phenotypically resistant to penicillin. These resistant isolates belonged to CC5, CC8, CC7, CC30, and CC97.

#### Tetracycline resistance

Two genes, *tet(38)* and *tet(K*), associated with tetracycline resistance were identified. The *tet(K*) gene was found in isolates exhibiting phenotypic resistance to tetracycline and these isolates predominantly belonged to the CC7 lineage. In contrast, the *tet(38*) gene was present across all 62 genomes and 59 isolates demonstrated susceptibility to tetracyclines.

#### Macrolide, Lincosamide, and Streptogramin (MLS) resistance

The primary genes associated with erythromycin resistance were *erm(T*) and *erm(A*). The *erm(T*) gene was identified in the *S. aureus* genomes of isolates from human swabs (CC398), whereas the *erm(A*) gene was identified in isolates from bulk tank milk (CC30). All these isolates were also phenotypically resistant to erythromycin. Additionally, five isolates (CC151) were phenotypically resistant to lincosamides, attributed to the presence of the *lnu(G*) gene.

#### Aminoglycoside resistance

For this category, the genes *aaC3* (found in all genomes) and *ant(9)-la* (identified in one genome from the CC30 complex) were the key markers. We only used gentamicin from the aminoglycosides class for phenotypic tests and did not find resistant isolates by disc diffusion methods.

##### Multidrug resistance efflux pumps

Our study identified nine widespread genes related to antibiotic efflux systems in all 62 genomes, including *lmrS, mepA, mepR, mepB, norA, norB, arlS, arlR, mgrA*. These genes contribute to resistance against diverse antibiotic classes, including fluoroquinolones, aminoglycosides, macrolides, and phenicols, amongst others. Moreover, the *mgrA* gene, a regulator for *norA* and *tet(38)*, was present in all genomes.

##### Fosfomycin resistance

The *fosB* gene was identified in 23 genomes conferring fosfomycin resistance. Phenotypic tests for fosfomycin were not conducted, as this is not a drug commonly used in dairy cattle. The *fosB* postive genomes belonged to isolates of CC5, CC8 and CC30. Point mutations related to fosfomycin resistance were identified, including: *murA*G257D in all CC8 isolates, three different point mutations (*murA* E291D, *glpT* L27F and *murA* T396N) in CC151 isolates, *glpT* V213I, *murA* D278E, and *murA* E291D in CC30 isolates, *glpT* F3I, *murA* D278E, and *murA* E291D in CC398 isolates, and *murA* E291D and *murA* T396N mutations in CC87 isolates.

##### Sulfonamides resistance

Resistance to sulfa drugs is mainly attributed to point mutations within the *folp* gene, which encodes the dihydropteroate synthase enzyme. We identified eight unique *folp* gene variants across the 62 isolates, with mutations scattered across 29 distinct amino acid sequence positions of DHPS (Table 2). We further noted that each of the eight clonal complexes was linked to a distinct *folp* variant (Table S8).

**Table 2.** Comparative analysis of amino acid variations at specific positions in DHPS protein sequence

	Position of amino acid that differs among the variant types in *folp* coding DHPS protein sequence for 62 isolates																												
**Variant Type**	**28**	**30**	**31**	**35**	**37**	**58**	**59**	**60**	**64**	**101**	**113**	**117**	**120**	**123**	**126**	**134**	**136**	**156**	**157**	**159**	**160**	**161**	**162**	**177**	**182**	**193**	**236**	**244**	**268**
**1**	S	I	N	A	I	V	S	L	M	M	R	I	K	A	I	N	D	I	A	I	P	S	N	N	V	E	R	E	L
**2**	S	V	T	A	M	I	T	V	L	I	R	V	K	A	V	N	D	I	A	I	P	S	N	N	I	E	R	E	L
**3**	S	V	T	A	I	V	S	L	M	I	H	I	K	A	I	N	D	I	A	I	P	S	N	N	V	E	R	E	L
**4**	S	I	N	T	I	V	T	L	L	I	H	I	K	A	V	N	D	I	A	I	P	S	N	N	V	E	R	E	L
**5**	S	V	T	A	M	I	S	L	L	I	R	V	K	S	V	N	D	I	A	I	P	S	N	N	V	E	R	E	L
**6**	T	I	N	A	I	V	T	L	L	M	R	V	K	A	I	N	D	I	A	I	P	S	N	N	V	E	R	E	L
**7**	S	V	T	A	M	I	T	V	L	I	R	V	K	A	V	N	D	I	A	I	P	S	N	N	V	E	R	E	F
**8**	S	V	T	A	M	V	S	L	L	I	H	V	N	A	V	E	E	M	S	V	S	V	D	E	V	G	K	A	L

## Discussion

Our investigation into the genomic epidemiology of *S. aureus*, collected from different sources on Vermont dairy farms, underscores the value of a hybrid assembly approach in determining nucleotide sequence-based STs and genetic markers of AMR. Our hybrid assembly pipeline uses the approach of assembling long read first and then polishing the assembly with short reads. The long-read-first assembly offers a robust strategy to achieve both structural accuracy and sequence completeness [46]. Using short reads for final polishing further refines the assembly, rectifying any lingering small-scale errors and optimizing the quality of the assembled genome [33,46].

### Genome assembly and annotation

Assessment of genome assembly completeness showed that the Hyb-Asm genome exhibited a higher completeness rate than the ONT-Asm or Illu-Asm genomes. This result agrees with the findings of Khezri *et al*. [17] who reported that hybrid assembly of four *Escherichia coli* and five *Klebsiella pneumoniae* clinical isolates performed better in completing the genomes compared to Nanopore sequenced long read assembly. Khezri *et al*. [17] also demonstrated the use of Flye for assembly of ONT long reads supporting our choice of genome assembly software tools. The fragmented genome assemblies generated from Illu-Asm were less contiguous than ONT-Asm and Hyb-ASM. Previous studies comparing the three assembly techniques reported fragmented assemblies limit the ability to determine the correct location of plasmids, large genomic rearrangements, and pan-genome analyses [47,48]. Our finding of more than a single contiguous sequence from the hybrid assembly approach is not unexpected, as some isolates of *S. aureus* will carry extrachromosomal plasmids, so we do not assume that the long read strategy failed to close the genomes of isolates with greater than one contiguous sequence. In future studies long read sequenced polished assemblies can be queried for using downstream Web tools such as PlasmidFinder (http://cge.cbs.dtu.dk/services/PlasmidFinder/), analogous to our downstream MLST typing and AMR detection approach. Across all three assembly approaches, read quality is paramount for achieving high fidelity in sequence prediction. Concurrently, sequencing depth plays a pivotal role in determining the contiguity of the assembled sequences. Thus, sequencing depth and read quality are crucial for achieving highly contiguous and accurate genome assemblies which is the strength of hybrid assembly [47,49].

### Strain typing

Using traditional Sanger sequencing as a gold standard for multilocus sequence typing, the hybrid assembly was more accurate in determining the MLST types of *S. aureus* isolates compared to ONT-Asm or Illu-Asm. This finding is consistent with a study where Chen *et al*. polished Oxford Nanopore long read assembled genomes with Illumina short reads to obtain accurate MLST prediction for a single *S. aureus* strain [20]. Another study by Tan *et al*. used ONT long reads to determine MLST type for *Streptococcus suis* isolates and reported that long read assembled genomes provide accurate MLST for eight out of ten isolates which improved after polishing [50]. Our results extend these prior findings by specifically applying the hybrid assembly approach to a panel of 62 *S*. *aureus* isolates. To the best of our knowledge this is the largest sample size of isolates where genomes generated from a hybrid assembly approach were used to predict the multilocus sequence type and compared to results of typing by traditional Sanger sequencing. Strengths of our study compared to previous work includes the sample size and our ability to demonstrate error rates of the ONT-Asm and Illu-Asm for MLST identification compared to Hyb-Asm. The discrepancies found in the MLST types from ONT-Asm genomes can be attributed to the higher error rate associated with long read sequencing [22]. In our analysis, we found that ONT-Asm genomes failed to provide accurate MLST predictions because of sequencing errors in homopolymers regions in the sequences of housekeeping genes. Multiple studies demonstrate that nanopore sequencers have difficulties in identifying the exact length of homopolymers [51,52]. Tan *et al*. also reported that the type of draft assembly polishing tool can affect the result of MLST from ONT-Asm genomes [50]. The discrepancies of MLST predictions for three isolates using Illu-Asm genomes in our study were attributed to the absence of hits for the genes *pta* and *glpF*. This discrepancy might be due to lower read depth of these three isolates ranging from 18× to 23× producing insufficient reads to assemble the absent gene sequences. A limitation of our study for comparing Illu-Asm results is the read depth which was optimized for use in polishing the ONT-Asm reads in the hybrid approach, and not for strain typing. We speculated that increasing Illu-Asm sequencing depth might improve MLST prediction accuracies when using this platform alone. Future studies might compare the cost-benefits of applying different sequencing platforms and bioinformatic pipelines in predicting accurate multilocus sequence types for epidemiological studies. For example, in some epidemiological contexts discriminating among clonal complexes may be sufficient and minor errors in ST prediction may be inconsequential.

The hybrid assembly and the ONT-Asm were concordant in their spa typing results and performed better than Illu-Asm. Since we did not perform PCR based spa typing for these isolates, we cannot validate the accuracy for the results of spa typing for the three pipelines. However, we included examples of duplicate isolates from the same source with the same MLST type, where Illu-Asm predicted different spa types, while Hyb-Asm and ONT-Asm gave consistent matching spa types. For instance, isolate AC6-122 and AC6-123 are the replicates of ST398 isolates collected from an individual hand swab, where Illu-Asm gave spa types t1250 and t571 respectively, while Hyb-Asm and ONT-Asm predicted t571 for both isolates. This suggests ONT-Asm and Hyb-Asm are superior to Illu-Asm for determining accurate *S. aureus* spa types. The discordant spa types from the Illu-Asm likely resulted from its short, fragmented reads and inability to resolve repetitive regions [20], which can produce truncated spa repeat profiles leading to erroneous spa types. Again, we speculate that increasing the coverage for Illumina short read sequencing may resolve some of these errors. In contrast, the long reads generated with Nanopore sequencing can span entire spa repeat units, enabling more accurate spa typing. These findings demonstrate the advantage of Hyb-Asm or ONT-Asm over short reads for whole genome typing *S. aureus* isolates using the repetitive spa locus.

One limitation of our current study comparing the three approaches is the difference in read depth we achieved for ONT and Illumina sequencing. We expect the errors we observed for Illu-Asm MLST typing to be corrected by sequencing of isolates with higher read depth. A recent paper suggested a mean depth of 100× to be sufficient for Illumina polishing the long-read assembly [46]. In our study we generated an average read depth of 27× from the Illumina platform, which reduced sequencing Illumina costs and was adequate for polishing ONT reads and correcting MLST error predictions. Beyond accurate predictions of strain types, long-read first hybrid assembly approaches appear to be preferred ‘state-of-the-art’ to generate error corrected complete bacterial genomes [46]. Alternatively, deeper sequencing using the Nanopore platform with new sequencing chemistries (e.g. R10.4.1 released in 2022) may eliminate or reduce the errors we observed with this technology, and perhaps eliminate the need for a hybrid approach [46]. By optimizing the sequencing depth on various platforms and leveraging the upcoming advancements in sequencing chemistries, we anticipate a potential reduction in the errors observed in our study. Researchers should identify the sequencing error rates that are acceptable within the context of specific research goals. For instance, in epidemiological studies, enhanced genetic resolution can markedly improve the identification of species or strains implicated in outbreaks and facilitate a deeper understanding of mutation rates, horizontal gene transfer, and transmission dynamics [53,54]. Enhanced resolution is also invaluable in tracing host switching events and in making informed mechanistic predictions about the functions of biosynthetic gene clusters [49]. The accuracy of genome assembly not only underpins fundamental genomic research but also extends to broader applications in public health and molecular biology.

### AMR genes identification

We observed concordant antimicrobial resistance gene identification across all assembly methods. It is also noteworthy that the WGS-based prediction of AMR based on ARG presence was largely consistent with phenotypic AST results, underscoring the potential of WGS in predicting AMR accurately. Whole genome sequencing has been used by several studies to assess the potential for predicting AMR genes from genomes of pathogens and validating concordances with the phenotypic results [19,55, 56]. In our study, we used five AMR databases to predict AMR genes to prevent the chance of missing any AMR acquired genes or chromosomal point mutation in the genes. The presence of beta lactam resistance genes, such as *blaz*, *blaI*, and *blaR*, along with tetracycline, macrolide, lincosamide, streptogramin, and aminoglycoside resistance genes in certain isolates highlights the extent of antimicrobial resistance in *S. aureus* populations across Vermont dairy farms. This finding is consistent with a previous study that reported similar antibiotic resistance gene profiles from different animals of New England [57]. The distribution of these AMR genes across different CCs reflects the widespread nature of these resistances. The inclusion of phenotypic data in our study underscores the notion that WGS-derived AMR gene identification offers predictive phenotypes. This was evident in our study when *tet(K*) was present in tetracycline-resistant isolates, whereas the susceptible isolates harboured the *tet(38)* gene.

Moreover, WGS predicted presence of *fosB*, a fosfomycin resistance gene and point mutations in genes contributing resistance to fosfomycin. We did not perform any phenotypic tests for fosfomycin resistance, however previous studies have used WGS and identified *fosB* and similar point mutation in *S. aureus* belonging to ST8, ST5, ST30 [56,58]. The *fosB* or related point mutations were not identified in CC97 isolates in our study which agrees with findings from another study [58]. We observed the presence of eight variants in the *folp* gene, related to sulfadimethoxine resistance, which has been reported previously [59]. Each variant was identified in a particular MLST CC of *S. aureus* which was also observed in the study by Nurjadi *et al*. where they identified associations between *folp* variants and MLST lineages [59].

In this study, we compared three assembly techniques only to identify the presence or absence of AMR genes rather than identifying the location of these genes. Previous studies have reported the successful use of hybrid assembly to not only identify the AMR genes and variants but also their location in the genome or mobile genetic elements for *E. coli* [60], *K. pneumoniae* [61]*,* and in mixed culture [17]. These findings lend credence to the superiority of hybrid assembly in yielding more complete and error free genome assemblies, making it an effective strategy for accurate AMR prediction of pathogens.

In conclusion, our results demonstrate that employing a hybrid assembly approach improved strain typing of *S. aureus* using whole genome MLST or spa typing. While all three assembly methods accurately predicted AMR genes, we expect that the hybrid approach offers the added advantage for studies with objectives to precisely identify AMR gene variants, determine the exact location of these genes, and ensure the completeness of the AMR gene operon. The continuous surveillance and analysis of genomic data together can be crucial to gain better insights into the changing landscape of antimicrobial resistance and strains of pathogens, which is fundamental for effective disease control strategies.

## supplementary material

10.1099/acmi.0.000766.v3 Uncited Supplementary Material 1.

## Abbreviations

AMR: antimicrobial resistance
ARGs: antibiotic resistance genes
CARD: comprehensive antibiotic resistance database
CC: clonal complex
DHPS: dihydropteroate synthase
Hyb-Asm: hybrid-assembled genomes
Illu-Asm: illumina-only assembled genomes
MIC: minimum inhibitory concentration
MLST: multilocus sequence typing
NCBI: National Centre for Biotechnology Information
ONT: Oxford Nanopore Technologies
ONT-Asm: ONT-only assembled genomes
SNPs: single nucleotide polymorphisms
spa: staphylococcal protein A
SRA: sequence read archive
ST: sequence type
WGS: whole-genome sequencing
wgSTs: whole-genome multilocus sequence types
